# PAMAM-cRGD mediating efficient siRNA delivery to spermatogonial stem cells

**DOI:** 10.1186/s13287-019-1506-4

**Published:** 2019-12-18

**Authors:** Tianjiao Li, Qiwen Chen, Yi Zheng, Pengfei Zhang, Xiaoxu Chen, Junna Lu, Yinghua Lv, Shiguo Sun, Wenxian Zeng

**Affiliations:** 1 Key Laboratory for Animal Genetics, Breeding and Reproduction of Shaanxi Province, Key Laboratory for Animal Biotechnology, Ministry of Agriculture of China, College of Animal Science and Technology, Northwest A&F University, Yangling, Shaanxi China; 2 Shaanxi Key Laboratory of Natural Products & Chemical Biology, College of Chemistry & Pharmacy, Northwest A&F University, Yangling, 712100 Shaanxi China

**Keywords:** PAMAM-cRGD, siRNA delivery, Nanoparticle, Spermatogonial stem cells

## Abstract

**Background:**

Spermatogonial stem cells (SSCs) are the cornerstone of sperm production and thus perpetual male fertility. In clinics, transplantation of patient’s own SSCs into testes is a promising technique to restore fertility when male germ cells have been depleted by gonadotoxic therapies. Auto-transplantation of genetically modified SSCs even has the potential to treat male infertility caused by genetic mutations. However, SSCs are refractory to transfection approaches. Poly(amidoamine) (PAMAM) dendrimers have the unique three-dimensional architecture, surface charge, and high density of surface groups that are suitable for ligand attachment, thereby facilitating target delivery. The goal of this study was to elucidate whether PAMAM dendrimers can efficiently deliver short interfering RNAs (siRNAs) to SSCs.

**Methods and results:**

We introduced cyclic arginine-glycine-aspartic acid (cRGD) peptides to the fifth generation of PAMAM dendrimers (G5) to generate PAMAM-cRGD dendrimers (G5-cRGD). The characterization of G5-cRGD was detected by Fourier transform infrared spectroscope (FTIR), transmission electron microscope (TEM), and the Cell Counting Kit-8 (CCK-8) assay. Confocal microscopy and flow cytometry were used to evaluate the delivery efficiency of siRNA by G5-cRGD to SSCs. The results showed that G5-cRGD encompassing siRNA could self-assemble into spherical structures with nanoscale size and possess high transfection efficiency, excellent endosomal escape ability, and low cytotoxicity, superior to a commercial transfection reagent Lipofectamine® 2000. Moreover, we demonstrated that G5-cRGD efficiently delivered siRNAs and triggered gene silencing.

**Conclusions:**

This study thus provides a promising nanovector for siRNA delivery in SSCs, facilitating the future clinical application of SSC auto-transplantation with genetically modified cells with a hope to cure male infertility that is caused by genetic disorders.

## Background

Spermatogonial stem cells (SSCs) are unique adult stem cells that transmit genetic information to subsequent generations. SSCs possess the potential of self-renewal and differentiation, thereby sustaining fertility throughout a man’s life [1, 2]. In clinics, auto-transplantation of genetically modified SSCs has the potential to treat male infertility and prevent transmission of genetic diseases to the offspring [3]. In addition, SSCs are able to generate donor-derived sperm after transplantation to a recipient’s testis [4]. Thus, genetic manipulation in combination with SSC transplantation provides a robust means to produce genetically modified offspring with improved productivity and production traits in animal industry [5].

However, studies and potential applications of SSCs have been greatly hampered due to low transfection efficiency. Previous reports have shown that SSCs are refractory to calcium phosphate and lipofection-mediated transfection [6, 7]. Meanwhile, electroporation typically yields significant cell death [8–10]. In case of viral transduction, viral sequences may integrate into the recipient genome, raising safety concerns in clinics [3, 11]. Therefore, there is an urgent need for development of novel strategies to deliver exogenous substances into SSCs with improved efficiency and safety.

Nanotechnology offers an attractive solution to the delivery problem. Among them, poly(amidoamine) (PAMAM) dendrimers are highly symmetric, spherical, hyper-branched, biocompatible, and nonimmunogenic [12, 13]. Importantly, PAMAM dendrimers exhibit high density of functional groups and high buffer capacity that facilitates endosome rupture and the release into cytoplasm [13, 14], making them as promising vectors for drug or gene delivery [13, 15]. Ziraksaz et al. reported that PAMAM could efficiently knock down octamer-binding transcription factor 4 (*Oct4*) gene expression in mouse embryonic stem cells [16]. However, cytotoxicity as well as poor targeting limits its application. Fortunately, modification with cyclic arginine-glycine-aspartic acid (cRGD) peptide reduces cytotoxicity, increases transfection efficiency in human anaplastic thyroid carcinoma (HTC/3) and malignant glioma (U87) cells [17, 18], and improves targeting ability [17, 19]. Hence, the objective of this study was to elucidate whether PAMAM dendrimers could efficiently deliver short interfering RNAs (siRNAs) to SSCs.

Here, we for the first time applied PAMAM-cRGD dendrimers (G5-cRGD) to SSC transfection in comparison with a commercial reagent Lipofectamine® 2000 (Lipo2000). We evaluated the physicochemical properties, cell toxicity, intracellular uptake, and gene silencing efficiency. We found that this vector was superior to Lipo2000 for delivery of siRNAs into SSCs. The outcome would contribute to studies on SSC biology and their potential applications to the clinics and animal production.

## Materials and methods

### Animals

ICR mice were purchased from the animal center of the Fourth Military Medical University, Xi’an, China. Testis samples were obtained from 6-day-old ICR mice. The experimental animals and procedures used in this study were approved by the Northwest A&F University’s Institutional Animal Care and Use Committee.

### Materials

The poly(amidoamine) (PAMAM) dendrimer with an ethylenediamine core (generation 5 with 128 surface amino groups) was purchased from CYD (Weihai, China, CYD-150A). Cyclic arginine-glycine-aspartic acid (cRGD) peptide was synthesized by GL Biochem Company (Shanghai, China, P170927-SY49331). 1-Ethyl-3-(3-dimethylaminopropyl) carbodiimide hydrochloride (EDC) and *N*-hydroxysuccinimide (NHS) were purchased from Sigma (St. Louis, MO, USA). The sequence of *Cdk1* siRNA: GCCAGATAGTGGCCATGAATT (21 bp), and the sequence of *Cdk2* siRNA: CUUCUAUGCCUGAUUAUAATT (21 bp). A scrambled siRNA duplex (21 bp) and FAM-labeled transfection scrambled siRNA (21 bp) were purchased from GenePharma (Shanghai, China). Lipofectamine® 2000 reagent was purchased from Invitrogen (Carlsbad, CA, USA, 11668019). All chemicals and reagents were of analytical grade.

### Preparation of G5-cRGD

1.2 μg of cRGD was dispersed in 10 ml phosphate buffer saline (PBS; pH = 7.4, 10 mM); then, 1.5 mg of EDC and 2.3 mg of NHS were added. The mixture was stirred for 1 h at 4 °C in the dark, followed by the addition of 5.7 mg PAMAM (G5). After 12 h of reaction, the resulted PAMAM-cRGD (G5-cRGD) was added to a dialysis bag (MwCO = 1000D) and incubated in 500 ml PBS (pH = 7.4, 10 mM) for 12 h at 4 °C in the dark. The final product was dried by a freeze-dryer.

### Structural characterization of G5-cRGD

The chemical structure of synthetic copolymers was characterized with Fourier transform infrared spectroscope (FTIR), specifically by VERTEX 70 FTIR Spectrometer (Bruker, Germany) in the range of 500–4000 cm^−1^. The samples were first mixed well with potassium bromide (KBr) and then compressed into a tablet for analysis.

### Cell isolation

The testis tissue was collected from 6-day-old ICR mouse pups. Testicular cells were obtained via a two-step enzymatic dissociation. In brief, testicular fragments were exposed to 1 mg/ml collagenase Type IV (Invitrogen, 17104019) for 5 min at 37 °C, followed by 0.25% trypsin-EDTA (Hyclone, Logan, UT, USA, SV30042.01) dissociation for 5 min. Single-cell suspension was prepared in DMEM/F12 medium (Hyclone, SH30023.01) containing 1% fetal bovine serum (FBS; Gibco, Grand Island, NY, USA, 10100147) and subjected to differential plating to remove the somatic cells [20]. To remove many peritubular myoid cells, the floating cells were transferred to a new plate after 0.5 h of incubation. Then, to remove Sertoli cells, the floating cells were transferred to a new plate after 2 h of incubation. Sertoli cells adhered to the plate and were maintained under the 37 °C with 5% CO_2_ of atmosphere. The floating cells which enriched with germ cell were cultured in CO_2_ incubator at 37 °C overnight.

### Purification of undifferentiated spermatogonia by fluorescent-activated cell sorting (FACS)

The uniform single-cell suspension after differential plating was used for cell sorting. After incubation with antibodies against E-cadherin (CDH1) for 30 min, cells were stained for 20 min on ice with anti-rabbit-Alexa Fluor 488. The cell fractions were washed with PBS and collected with a FACS Aria III cell sorter (BD Biosciences). The finally acquired CDH1^+^ germ cells were used for primary culture.

### Cell culture

The C18-4 cell line was established from undifferentiated type A spermatogonia [21] and obtained from Dr. Zuping He at Shanghai Jiao Tong University, China. The cells were validated using various markers for mouse germ cells and SSCs [22]. The cells were cultured in DMEM/F12 containing 10% FBS (BI, Israel, 04-121-1A) and 100 unit/ml penicillin and streptomycin (Invitrogen, 15140122) and maintained at 37 °C in a humidified incubator (5% CO_2_). The C18-4 cell line was characterized by using different SSC markers (CDH1 and Lin28) as shown in Additional file 1: Figure S2A.

The enriched SSCs were cultured in DMEM-F12 supplemented with 2% FBS (Gibco), 5% knockout serum replacement (KSR; Gibco, 10828028), 100 mM nonessential amino acid solution (Invitrogen, 11140050), 100 unit/ml penicillin and streptomycin (Invitrogen, USA), 2 mM l-glutamine (Invitrogen, 25030081), 1 × MEM vitamin (Invitrogen, 11120052), 30 ng/ml β-estradiol, 10 ng/ml Glial cell line-derived neurotrophic factor (GDNF), and 10 ng/ml bFGF. The isolated Sertoli cells were validated using the specific marker (SOX9) as shown in Additional file 1: Figure S5. The cells were maintained in DMEM/F12 medium with 5% FBS and cultured for three to four passages. To prepare feeder cell monolayers, Sertoli cells were mitotically inactivated by treatment with mitomycin C (10 mg/ml) for 3 h followed by extensive washing in DPBS.

### Transmission electron microscope (TEM)

G5-siRNA complexes (dendriplexes) and G5-cRGD-siRNA complexes (cRGD-dendriplexes) with the ratio of nitrogen atoms in the dendrimer to phosphorous atoms in the siRNA (N/P ratio) of 10 were measured by a transmission electron microscope (TEM; FEI, USA). The prepared specimens were diluted and added to a drop of the polyplex solution on a copper grid to dry naturally. The dried specimens were observed through a transmission electron microscope at an accelerating voltage of 80 kV.

### Preparation of nano-siRNA complexes and detection of compensation efficiency

Nano-siRNA complexes containing dendriplexes and cRGD-dendriplexes were prepared by mixing scramble siRNA and cationic dendrimers in serum-free Opti-MEM medium (Gibco, 31985062) at various N/P ratios from 0 to 40 at 37 °C for 30 min. Twenty microliters of the complexes containing the 0.1 μg of siRNA solution was electrophoresed on the 3.5% agarose gel containing ethidium bromide at 120 V for 20 min. A free naked siRNA was used as a negative control. The bands of siRNA were visualized under ultraviolet (UV) illumination condition.

### Zeta potential measurement

Zeta potential of the nanoparticles and nano-siRNA complexes were measured using a Mastersizer ZEN3600 (Malvern Instruments Ltd., Malvern, UK). Nano-siRNA complexes were prepared at different N/P ratios and then diluted with DPBS to 1.0 ml volume before measure. Each sample was done in triplicate, and the average value was used. Reported values were calculated as the average of three independent experiments comprising 3 measurements for each.

### Immunocytochemistry

Cells were seeded on 96-well plates and treated with nano-siRNA complexes, fixed with 4% PFA for 20 min at room temperature, and permeabilized for 10 min using 0.1% Triton X-100 solution. Blocking was performed to suppress non-specific antibody binding by incubation with 10% donkey serum (Abcam, Cambridge, UK, ab7475) for 2 h. Subsequently, the cells were incubated with the primary antibodies against THY1 (1:200; Santa Cruz Biotechnology, CA, USA, SC-9163), CDH1 (1:200; Proteintech, Chicago, IL, USA, 20874-1-AP), PLZF (1:100; Invitrogen, USA, PA5-29213), Lin28 (1:200; Abcam, Cambridge, UK, ab46020), or SOX9 (1:200; Santa Cruz Biotechnology, CA, USA, SC-166505) at 4 °C overnight. Afterwards, samples were incubated with the corresponding secondary antibody anti-mouse-Alexa Fluor 594 (1:200; Jackson Immunoresearch, 715-585-151), anti-rabbit-Alexa Fluor 488 (1:300; Jackson Immunoresearch, 711-545-152), or anti-rabbit-Alexa Fluor 594 (1:300; Jackson Immunoresearch, 711-585-152) at room temperature for 1 h and counterstained with Hoechst 33342 (Beyotime Institute of Bio-technology, C1022). Negative controls were incubated with 10% donkey serum without the primary antibody. Finally, fluorescent images were acquired with an Olympus IX71 (Tokyo, Japan) inverted fluorescence microscope camera.

### Cytotoxicity assay

Cell viability was determined by Cell Counting Kit-8 (CCK-8) assay (Beyotime Institute of Biotechnology, Jiangsu, China, C0042) and MTT assay (Beyotime Institute of Biotechnology, Jiangsu, China, C0009). The cells were prepared and dispersed in 96-well cell culture plates at a cellular density of 5.0 × 10^3^ cells/well and cultured for 24 h. In order to detect the toxicity of different concentrations of nanoparticle (NPs), the cells were treated with G5 and G5-cRGD NPs, respectively, at various concentrations (10, 20, 30, 40, and 60 μg/ml) for 24 h at 37 °C. In addition, to detect the toxicity of different N/P ratios of nano-siRNA complexes, the cells were transfected with dendriplexes and cRGD-dendriplexes, respectively, at various N/P ratios for 48 h, followed by incubation with Opti-MEM medium only for 6 h. Finally, 10 μl of CCK-8 solution in DMEM was added to each well and incubated at 37 °C for 2 h. The optical density of each well was measured at 450 nm with a microplate reader. For the MTT assay, 10 μl of MTT (5 mg/ml) reagent was added to each well and incubated for 4 h. Formazan solvent (100 μl) was added to each well until all crystals dissolved. The absorbance was measured at 570 nm by a microplate reader. Results were calculated as the absorbance of treated cells relative to untreated controls. At least three independent experiments were performed.

### Cell transfection

Before transfection, 1.5 × 10^5^ cells were seeded on plates (6-well) with fresh complete DMEM medium. The commercial Lipofectamine® 2000 reagent (Lipo2000) was used according to the manufacturer’s instruction. Dendriplexes and cRGD-dendriplexes were assembled at N/P ratios ranging from 1 to 15 in serum-free Opti-MEM medium at 37 °C for 30 min. The final siRNA concentration was adjusted to 100 nM with complementing Opti-MEM medium. After 6 h of incubation, the transfection mixture was replaced with the complete medium and maintained for further incubation up to 48 h. A free naked siRNA was used as a negative control. In order to facilitate the observation of cellular internalization, we used FAM-labeled siRNA for transfection. The cellular internalization was observed under a REVOLUTION WD confocal laser scanning microscope (CLSM; Andor, UK) with a 100 × oil immersion lens.

### Flow cytometry

To determine the siRNA delivery efficiency, cells were transfected with FAM-labeled siRNA at 100 nM. The cells were seeded on 6-well culture plates for 24 h, and the medium was then replaced with the control (Opti-MEM), free siRNA, dendriplexes, and cRGD-dendriplexes at various N/P ratios, respectively. After 6 h of incubation in serum-free Opti-MEM medium, the cells were trypsinized and were re-suspended in 100 μl of cold PBS. Naked siRNA was used as a negative control. A total of 10,000 events were collected for each measurement. The transfection efficiency was calculated by the percentage of GFP^+^ cells. FlowJo software was used to analyze the data and quantify the mean fluorescence intensity (MFI) per cell. Each assay was performed in triplicates.

### Uptake pathway studies by inhibiting endocytosis

To inhibit the endocytic pathways, cells were treated with sodium azide (NaN_3_) and under low-temperature condition, respectively. The cells were pre-incubated with NaN_3_ (40 μM) for 30 min, followed by incubation at 37 °C with cRGD-dendriplexes (N/P 10:1) for 2 h. Later, blocking experiments were carried out by incubating cells with cRGD-dendriplexes at 4 °C for 1 h. Finally, the samples were washed three times with PBS, and fluorescent micrographs were acquired with an Olympus IX71 (Tokyo, Japan) inverted fluorescence microscope camera.

### Endosomal escape

Cells were seeded at a density of 5 × 10^4^ cells/ confocal dish and incubated for 48 h. The cells were incubated with dendriplexes or cRGD-dendriplexes (N/P 10:1) for 6, 12, 24, and 36 h, respectively. Subsequently, the cells were treated with Lyso-Tracker Red (Beyotime Institute of Bio-technology, C1046) for 1 h and stained with Hoechst 33342 (Beyotime Institute of Bio-technology, C1022) for 20 min at 37 °C. Finally, fresh culture medium without phenol red was added. The samples were captured by REVOLUTION WD CLSM (Andor) with a 100 × oil immersion lens. Quantitative colocalization showed the proportion of the FAM-labeled siRNA that colocalize with Lyostracker Red (yellow puncta) to the FAM-labeled siRNA (green puncta) per cell. Endosomal escape experiments were repeated three times and counted 100 cells.

### Quantification of germ cell colonies

Primary SSC-derived colonies were assessed by the result described by Kanatsu-Shinohara et al. with a minor modification [20]. CDH1-FACS sorted germ cells were seeded at 2 × 10^4^ cell/well in 48-well dishes on feeder layers (Sertoli cells). To count colonies, the cells were cultured for 5 days after transfection with nano-siRNA complexes for 12 h. The morphology of cell colonies was observed by inverted microscope (Olympus IX71, Tokyo, Japan). Next, we calculated the mean number of colonies (a cluster consists of more than 5 cells) per square centimeter. Experiments were repeated three times.

### Proliferation assay

To assess proliferation, the cells were seeded on 96-well plates. After transfection, cell proliferation was detected using an EdU Cell Proliferation Assay Kit (Ribobio, Guangzhou, China, C10310-3). The EdU assay was performed 2 or 7 days after transfection in SSC line and primary SSCs, respectively. In brief, cells were maintained in complete medium with 50 μM of EdU for 2 to 4 h. After fixation with 4% paraformaldehyde (PFA) for 30 min at room temperature, the cells were neutralized by glycine (2 mg/ml). After washing, the cells were permeabilized with 0.5% Triton X-100 solution. The EdU staining was performed. Subsequently, cells were stained with Hoechst 33342 and evaluated under a fluorescent microscope (Olympus, Tokyo, Japan). Cell proliferation activity was determined by defining the proliferation index: the ratio of EdU-positive cells to total number of cells. Experiments were repeated three times, and each time we randomly selected 5 fields for statistics. A total of 500 cells were counted.

### Cell cycle assay

Cell cycle progression was examined by flow cytometer using 4′,6-diamidino-2-phenylindole (DAPI) staining. Cells were seeded in six-well culture plates. Cell cycle assay was performed 7 days after transfection in primary SSCs. The cells were stained with CDH1 using anti-rabbit-Alexa Fluor 488. Subsequently, cell cycle was monitored by using DAPI staining of nuclei and subjected to flow cytometry analysis [23]. All measurements analysis was carried out using ModFit (Verity Software, Topsham, ME, USA) data analysis software. Experiments were performed independently at least three times.

### TUNEL assay

To detect cell apoptosis, the cells were seeded on 96-well plates. After transfection, cell apoptosis was detected using a TUNEL BrightRed Apoptosis Detection Kit (Vazyme, Jiangsu, China, A113-01). The TUNEL assay was performed 7 days after transfection in primary SSCs, following the manufacturer’s instructions. In brief, cells were fixed in 4% paraformaldehyde and permeabilized for 10 min using 0.1% Triton-X100 solution at room temperature. Then, the cells were incubated with 50 μl TdT incubation buffer at 37 °C for 1 h in darkness. Finally, cells were stained with DAPI and evaluated under a fluorescent microscope (Olympus, Tokyo, Japan). The ratio of the TUNEL-positive cells (red fluorescence) to the CDH1-positive cells (green fluorescence) indicated the primary SSC apoptosis. Experiments were repeated three times, and each time we randomly selected 5 fields for statistics.

### RNA extraction and quantitative real-time PCR analysis

RNA was extracted from the cells with Trizol reagent (Takara, 9109) according to the manufacturer’s protocol. For each sample, 1 μg of total RNA was subjected to reverse transcription using Transcriptor First Strand cDNA Synthesis Kit (Roche, Mannheim, Germany, 04897030001). FastStart Universal SYBR Green Master (Roche, 38697500) was used for Real-time PCR using an IQ5 (Bio-Rad). *β-actin* and GAPDH were used as the internal reference, and the data were analyzed using the 2^−∆∆CT^ method [24].

### Western blot

Proteins were extracted using RIPA buffer (Solarbio, Beijing, China, R0020) according to the manufacturer’s protocol. The concentration of protein was measured by BCA kit (Takara, Japan, AI90451A). Thirty micrograms of total proteins from each sample were separated by polyacrylamide gel electrophoresis and transferred onto poly-vinylidene fluoride (PVDF) membranes (Millipore, Billerica, MA, USA). The blot-transferred membranes were blocked with 5% fat-free dry milk for 2 h and incubated with primary antibodies: mouse anti-PCNA (1:500; Santa Cruz Biotechnology, PC10), mouse anti-CDK1 (1:500; Santa Cruz Biotechnology, AN21.2), mouse anti-CDK2 (1:500; Santa Cruz Biotechnology, D-12), and mouse anti beta-ACTIN (1:2000; CWBIO, Beijing, China, CW0096) on a shaker at 4 °C overnight, followed by incubation with horseradish peroxidase (HRP)–conjugated anti-mouse IgG (1:2000; Millipore, AP192P). Protein bands were visualized and captured by a Bio-Rad Chemidoc XRS using a Western Bright ECL Kit (Bio-Rad, Berkeley, CA, USA). The protein bands were hand-drawn for 3 times and calculated the average intensity by Image-Pro Plus. The ratio of the target protein to the corresponding intensity value of β-actin was obtained for analysis. Experiments were repeated three times.

### Statistical analysis

The data obtained are shown as mean ± standard deviation (SD). One-way ANOVA or Student’s *t* test was performed to determine the differences of the data. A value of *P* < 0.05 was considered statistically significant.

## Results

### Synthesis and characterization of G5-cRGD and cRGD-dendriplexes

Based on the previous report [25], the cRGD was modified on the surface of the fifth generation of PAMAM dendrimers (G5). The structural composition of PAMAM-cRGD dendrimers (G5-cRGD) was confirmed by FTIR. The weak characteristic peak of sulfhydryl group (−SH) appeared at 2500 cm^−1^ indicates the successfully modification of cRGD on the surface of PAMAM (Additional file 1: Figure S1).

**Fig. 1:**
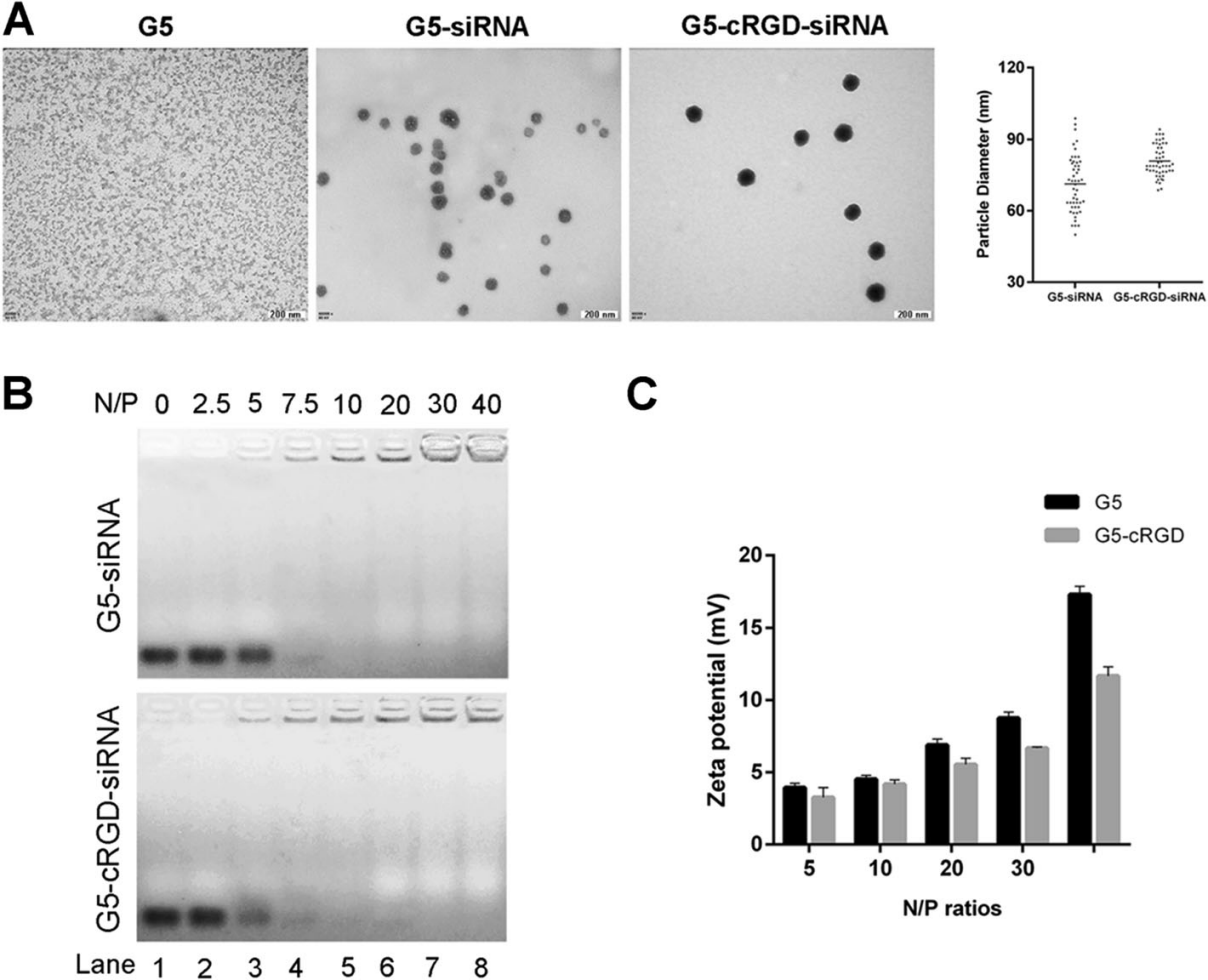
Physicochemical characterization of nano-siRNA complexes. **a** TEM images of G5 and the complexes of G5-siRNA (dendriplexes) and G5-cRGD-siRNA (cRGD-dendriplexes) at the N/P ratio of 10. Scale bar is 100 nm. Particle size distribution of nano-siRNA complexes obtained by TEM images. Fifty nanoparticles were counted. **b** Representative images of gel retardation assay for G5-siRNA and G5-cRGD-siRNA at different N/P ratios. Lane 1: naked siRNA, lanes 2–8: nano-siRNA complexes (N/P ratios 2.5:1, 5:1, 7.5:1, 10:1, 20:1, 30:1 and 40:1). **c** Zeta potentials of nano-siRNA complexes at different N/P ratio

The size and morphology of nanoparticles were determined by TEM. The G5 nanoparticles were in irregular shapes (Fig. 1a). Upon addition of siRNA to G5 or G5-cRGD, these nano-siRNA complexes self-assembled into spherical structures with the average particle sizes of 71.37 and 80.86 nm, respectively (Fig. 1a). Interestingly, the G5-cRGD-siRNA complexes (cRGD-dendriplexes) were more uniform than G5-siRNA complexes (dendriplexes).

To determine the load efficiency of siRNA in the nano-vectors, the agarose gel retardation assay was performed. With the increase of N/P ratios, siRNA bands gradually decreased and completely disappeared when the N/P ratio was higher than 10 in either dendriplexes or cRGD-dendriplexes (Fig. 1b), indicating that G5 and G5-cRGD are able to efficiently bind and load siRNAs. As shown in Fig. 1c, G5 nanoparticle exhibited positive zeta potential values around + 17.30 mV, while G5 modification with cRGD could partially reduce the cationic charge. Zeta potentials of the nano-siRNA complexes gradually increased with increasing N/P ratio.

### Cytotoxicity assay of G5-cRGD in the C18-4 undifferentiated spermatogonial cell line

**Fig. 2:**
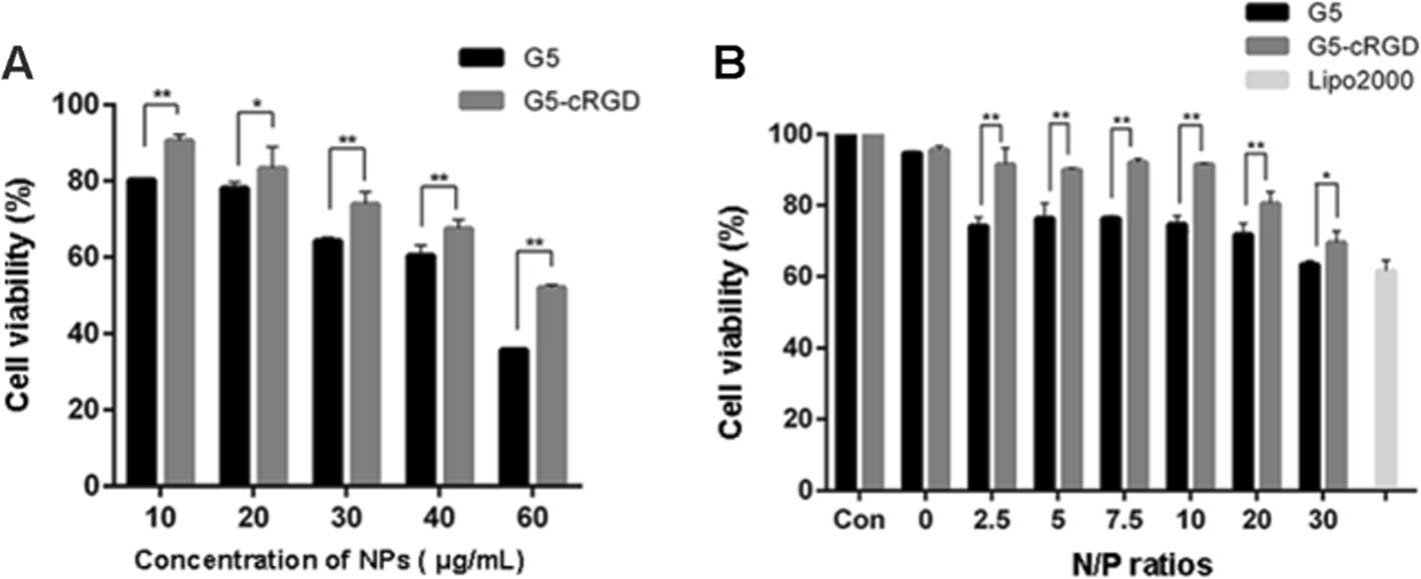
Toxicity profile of the G5-cRGD-mediated delivery system. **a** The viability of the SSC line incubated with G5 and G5-cRGD nanoparticles at various concentrations by CCK-8 assay. **b** The viability of the SSC line transfected with G5-siRNA and G5-cRGD-siRNA complexes at different N/P ratios for 24 h, as evaluated by CCK-8 assay. The final siRNA concentration of each sample was 100 nM. Lipo2000 was used as a positive control. Statistical significance was determined by applying the Student’s *t* test. Data are presented as mean ± standard deviation (SD, *n* = 3). *p < 0.05, **p < 0.01

The mouse SSC line C18-4 cells were characterized by using different SSC markers. Immunocytochemical staining revealed that CDH1 [26] and Lin28 [27] were expressed in C18-4 cells (Additional file 1: Figure S2A). Subsequently, the cytotoxicity of G5, G5-cRGD, dendriplexes, and cRGD-dendriplexes was evaluated in an SSC line by CCK-8 assay. Cell viability decreased with the increase of the concentration of nanoparticles. The viability in G5-cRGD group was higher than that in G5 (Fig. 2a). The viability also reduced with the increase of charge ratio (Fig. 2b). It was higher in the G5-cRGD group than in the G5 group in all the ratios tested. MTT assay was used to corroborate these observations (Additional file 1: Figure S2B and C). Importantly, cell viability in G5-cRGD group was over 90% with a 10:1 ratio, which represented a good biocompatibility. Cell viability in the positive control, a commercial reagent Lipo2000, was 60.31% (Fig. 2b). These results demonstrate that conjugation of G5 with cRGD leads to less cytotoxicity.

### Transfection efficiency and uptake pathway of G5-cRGD in the C18-4 undifferentiated spermatogonial cell line

**Fig. 3:**
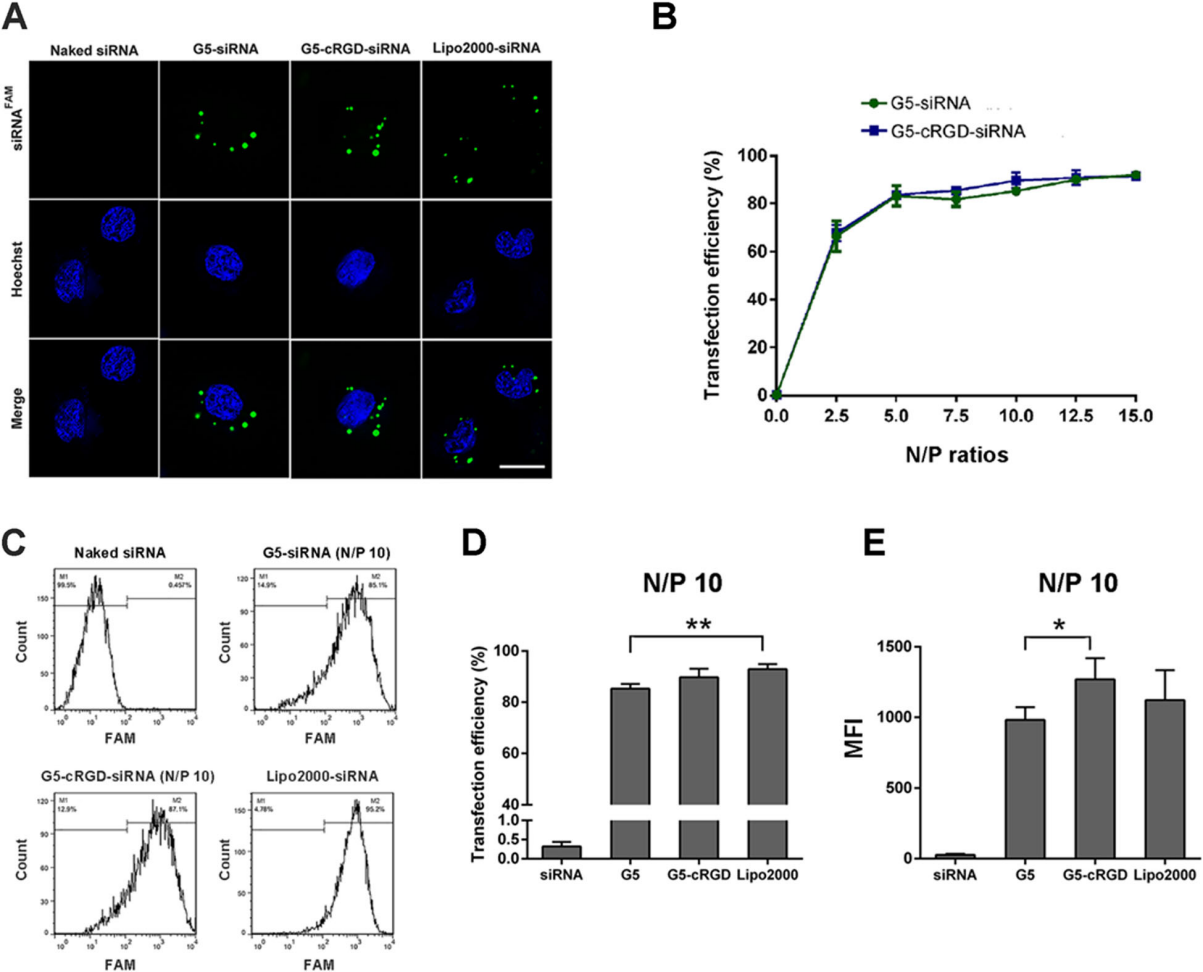
G5-cRGD-mediated siRNA delivery in an SSC line. **a** Intracellular localization of nano-siRNA complexes by confocal laser scanning microscope (CLSM). Images were taken when cells were incubated for 6 h. The nuclei were stained blue by Hoechst 33342, and the green spots refer to internalized nano-siRNA complexes. The scale bar is 10 μm. **b** Transfection efficiencies of nano-siRNA complexes at various N/P ratios by flow cytometry after 6 h of incubation. Flow cytometry histograms (**c**) and bar chart (**d**) indicate the transfection efficiency of different FAM-siRNA formulations at the N/P ratio of 10. **e** Mean fluorescence intensity (MFI) of G5, G5-cRGD, and Lipo2000 with FAM-siRNA (N/P 10:1) determined by flow cytometry. Statistical significance was assessed using one-way ANOVA followed by Dunnett’s test. Data are presented as mean ± standard deviation (SD, *n* = 3). *p < 0.05, **p < 0.01

Live cell confocal imaging was performed to visualize the intracellular distribution of nano-siRNA complexes. The fluorescence was localized in the cytoplasm when cells were treated with dendriplexes or cRGD-dendriplexes (Fig. 3a), indicating that siRNAs are effectively delivered into SSCs via G5 and G5-cRGD.

Higher N/P ratios are essential to break the cell membrane barrier and improve transfection efficiency, which is, in turn, accompanied by damages to cell membrane integrity. The transfection efficiency increased with the increase of N/P ratio (Fig. 3b). The percentage of FAM-positive cells was more than 80% when the N/P ratio was 5, and reached up to 90% when N/P was 10. Further increase N/P ratio did not yield higher transfection efficiency. Taken together the data from the gel retardation assay and cytotoxicity test (Figs. 1b and 2b), we decided to use the N/P ratio of 10 in the following experiments. When the N/P ratio was 10, transfection efficiency in G5-cRGD group (89.73 ± 3.34%) was similar with in Lipo2000 (92.84 ± 1.95%), in G5 (85.33 ± 1.76%) was less than in Lipo2000 (Fig. 3c, d). In addition, flow cytometry assay showed that mean fluorescence intensity (MFI) in G5-cRGD was significantly stronger than that in G5 group (Fig. 3e). It has been reported that higher MFI could lead to higher targeted delivery efficiency [28].

We determined by qualitative analysis on immunofluorescent images whether uptake depends on active translocation. The fluorescence in G5-cRGD-siRNA group was stronger than in G5 group (Additional file 1: Figure S3C and F). Sodium azide (NaN_3_), an oxidative phosphorylation inhibitor, is able to abolish ATP production within the membrane and prohibit endocytosis [29]. Interestingly, the fluorescence intensity decreased in NaN_3_ treatment although it was still discernable (Additional file 1: Figure S3I). Moreover, the cellular uptake of cRGD-dendriplexes significantly reduced at 4 °C in comparison with the control at 37 °C (Additional file 1: Figure S3L).

### Endosomal escaping of siRNA in the C18-4 undifferentiated spermatogonial cell line

Efficient uptake of siRNA is the first step for gene silencing, and the successful escape of siRNA from endosomes into the cytoplasm is equally important [13]. To detect the endosomal escaping, we stained endosome/lysosomes with Lyso-Tracker Red. In the confocal images, yellow fluorescence represented the colocalization of FAM-siRNA (green) with late endosomes/lysosomes (red). The yellow fluorescence revealed that cRGD-dendriplexes distributed in endosomes/lysosome at 6 h post transfection, and more yellow spots localized in the cytoplasm at 12 h. After 24 h, a small number of green spots separated from Lyso-tracker Red, indicating that siRNA began to escape from the endosomes (Additional file 1: Figure S4A). More green fluorescence was separated from the red fluorescence at 36 h (Additional file 1: Figure S4B). The co-localization ratio significantly decreased to 47.20% in the cRGD-dendriplexes group, while 66.81% in the dendriplexes group at 36 h (Additional file 1: Figure S4C).

### Functional siRNA delivery in the C18-4 undifferentiated spermatogonial cell line

**Fig. 4:**
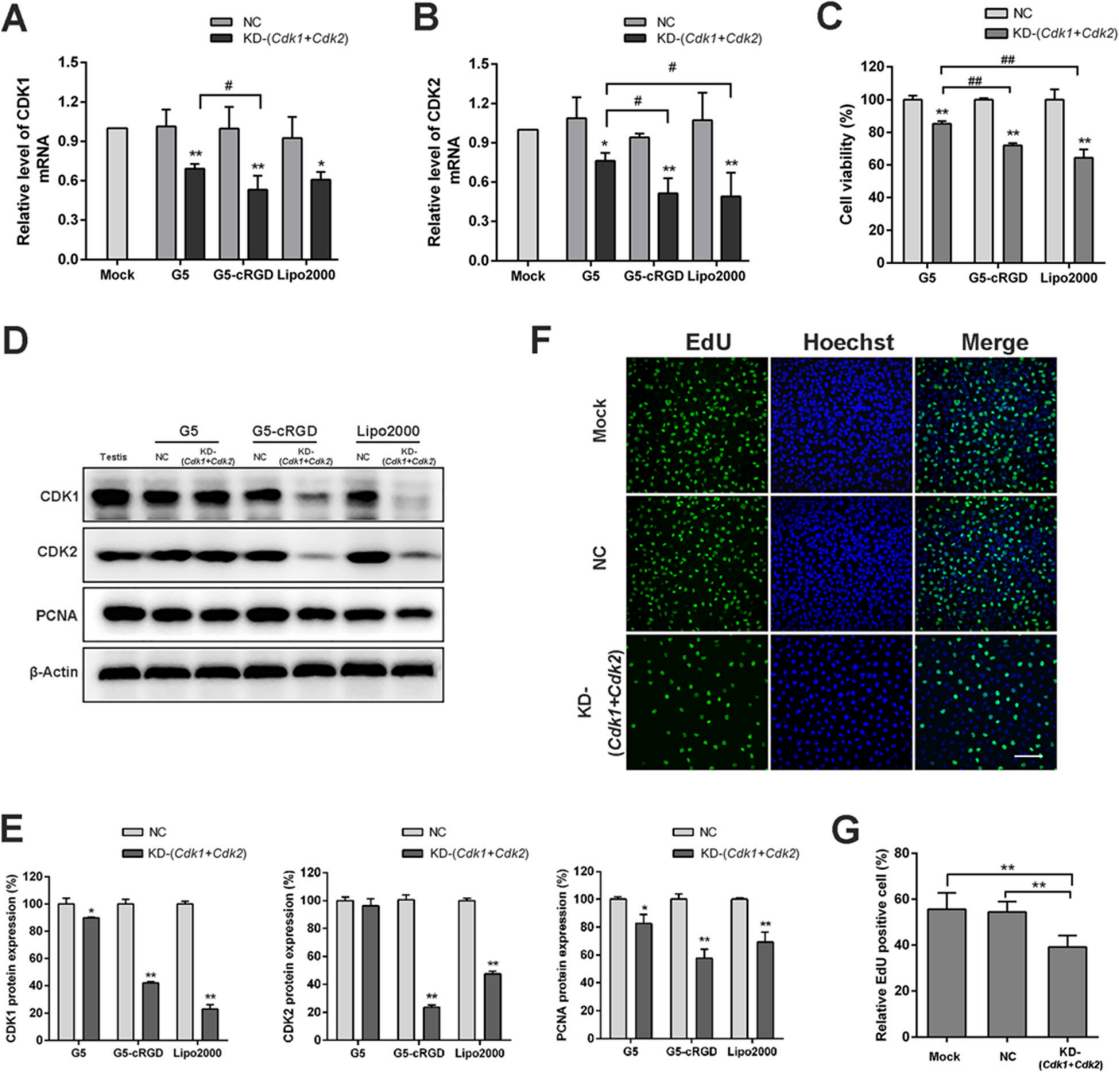
Gene silencing efficiency and cell proliferation assay in C18-4 cell line. The Relative *Cdk1* (**a**) and *Cdk2* (**b**) mRNA expression after treatment with siRNA against *Cdk1* (50 nM) and *Cdk2* (50 nM) compared with the Mock group in cells at the N/P ratio of 10. Lipo2000 was used as a positive control. **c** Cell viability was assessed by the CCK-8 assay at 48 h post-treatment. **d** CDK1 and CDK2 level was detected by Western blot. The expression of PCNA at the protein level indicates the status of proliferation. Positive control: whole testes lysates. **e** Intensity analysis of CDK1, CDK2, and PCNA were calculated by Image-Pro Plus. **g** Bar graphs of cell multiplication upon (**f**) EdU incorporation assay were shown as the ratio of EdU-positive cells. Scale bars: 100 μm. Statistical significance was assessed using one-way ANOVA or Student’s *t* test. Data are presented as mean ± standard deviation (SD). *p < 0.05, **p < 0.01 compared with NC in the same vector group; ^#^*p* < 0.05, ^##^*p* < 0.01 compared with KD-(*Cdk1+Cdk2*) in different vector group. Mock: untreated group; NC: scramble siRNA group; KD-(*Cdk1+Cdk2*): *Cdk1+Cdk2* siRNA group

Cyclin-dependent kinases (Cdks) 1 and 2 are involved in the regulation of cell cycle progression. To assess the nanoparticle delivery system, SSCs were treated with cRGD nanoparticles carrying siRNA against *Cdk1 and Cdk2.* We detected whether knockdown of *Cdks* by G5-cRGD-mediated siRNA delivery is associated with disturbances in cell cycle progression. As shown in Fig. 4a, b, expression of *Cdk1* and *Cdk2* was significantly lower in knockdown groups than that in NC groups, suggesting that both *Cdk1* and *Cdk2* siRNAs worked in G5, G5-cRGD, and Lipo2000 approaches. Moreover, the expression of CDK1 and CDK2 in KD-(*Cdk1+Cdk2*) group was significantly decreased at the protein level when G5-cRGD or Lipo2000 was used as a carrier (Fig. 4d, e). In line with *Cdks* expression, cell viability in G5-cRGD was significantly lower than that in G5 vector (Fig. 4c). Importantly, expression of proliferating cell nuclear antigen (PCNA) in G5-cRGD was lower than that in G5 cargo with siRNAs (Fig. 4d, e). Subsequently, EdU assay further showed that delivery of siRNAs with G5-cRGD led to a significant decrease of EdU-positive cells (Fig. 4f, g).

### Transfection efficiency of G5-cRGD in primary SSCs

**Fig. 5:**
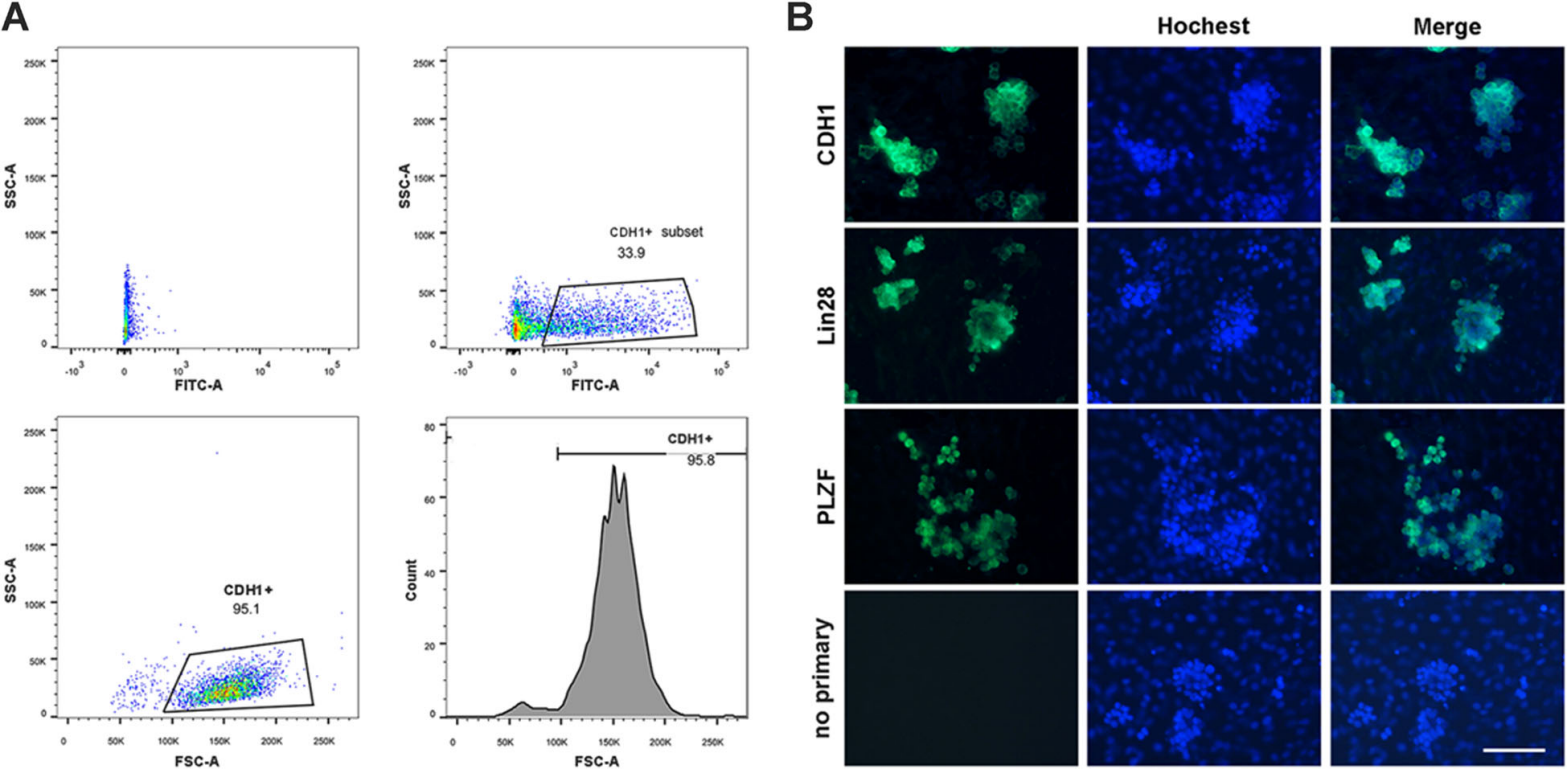
Purification and characterization of primary SSCs. **a** Flow cytometry data to illustrate the gating strategy for FACS purification of CDH1^+^ germ cells. **b** Immunocytochemical staining of SSC colonies on Sertoli cell feeder layer after 14 days of culture. The fluorescence staining is performed on CDH1 FACS sorted cells following differential plating. Expression of CDH1, Lin28, and PLZF in SSC colonies was showed in green fluorescence. Negative (no primary) control: omission of primary antibody. The nuclei (blue) were stained with Hoechst 33342. Scale bar: 100 μm

Analysis of FACS-sorted cells showed that E-cadherin (CDH1)-positive cells reached 95% (Fig. 5a). Immunocytofluorescence analysis showed that almost all colonies were positive for CDH1, promyelocytic leukemia zinc-finger (PLZF) [30], and lin-28 homolog A (Lin28), indicating the SSC origin (Fig. 5b).

**Fig. 6:**
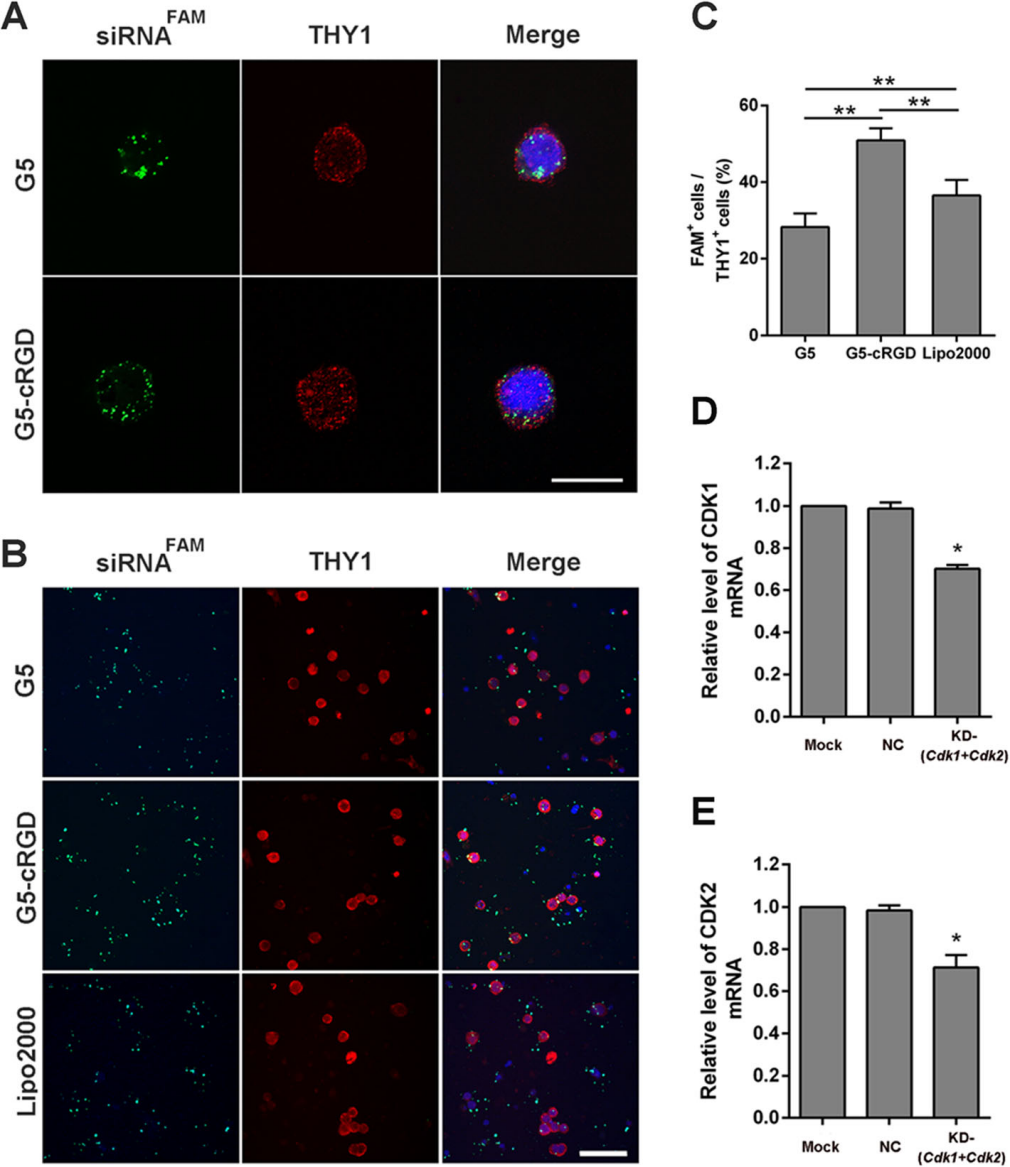
G5-cRGD-mediated siRNA delivery in primary SSCs. **a** Localization of FAM-siRNA with G5 or G5-cRGD as carries was examined by CLSM 12 h after the transfection. Labeled siRNA was located in the THY1-positive cell cytoplasm. Red: THY1 staining used to mark SSCs; green: FAM-labeled siRNA; blue: Hoechst 33342 used to stain nuclei. Scale bars: 10 μm. **b** Fluorescent photographs were prepared 12 h after transfection with different FAM-siRNA formulations. Scale bars: 50 μm. **c** Percentages of FAM-positive cells among the THY1-positive cells were determined from 20 randomly selected regions by three independent experiments. Suppression of *Cdk1* (**d**) and *Cdk2* (**e**) using the G5-cRGD delivery vector was examined in primary SSCs at the mRNA level. Statistical significance was assessed using one-way ANOVA followed by Dunnett’s test. Data are presented as mean ± standard deviation (SD). *p < 0.05, **p < 0.01. Mock: untreated group; NC: scramble siRNA group; KD-(*Cdk1+Cdk2*): *Cdk1+Cdk2* siRNA group

Subsequently, we investigated the potential application of G5-cRGD for siRNA transfection in primary SSCs. First, localization of FAM-labeled siRNA was examined by confocal microscopy. CDH1-FACS-sorted germ cells were stained for Thymocyte antigen 1 (THY1; a SSC marker). As shown in Fig. 6a, labeled-siRNAs were observed in the cytoplasm of THY1-positive cells. There were 28.28 ± 3.33% THY1-positive cells displaying FAM fluorescence in the G5 group, while the percentage increased to 50.92 ± 3.17% in the G5-cRGD group. More importantly, the transfection efficiency in G5-cRGD was also prominently improved in comparison with Lipo2000 (36.31 ± 4.07%; Fig. 6b, c).

### Functional siRNA delivery in primary SSCs

**Fig. 7:**
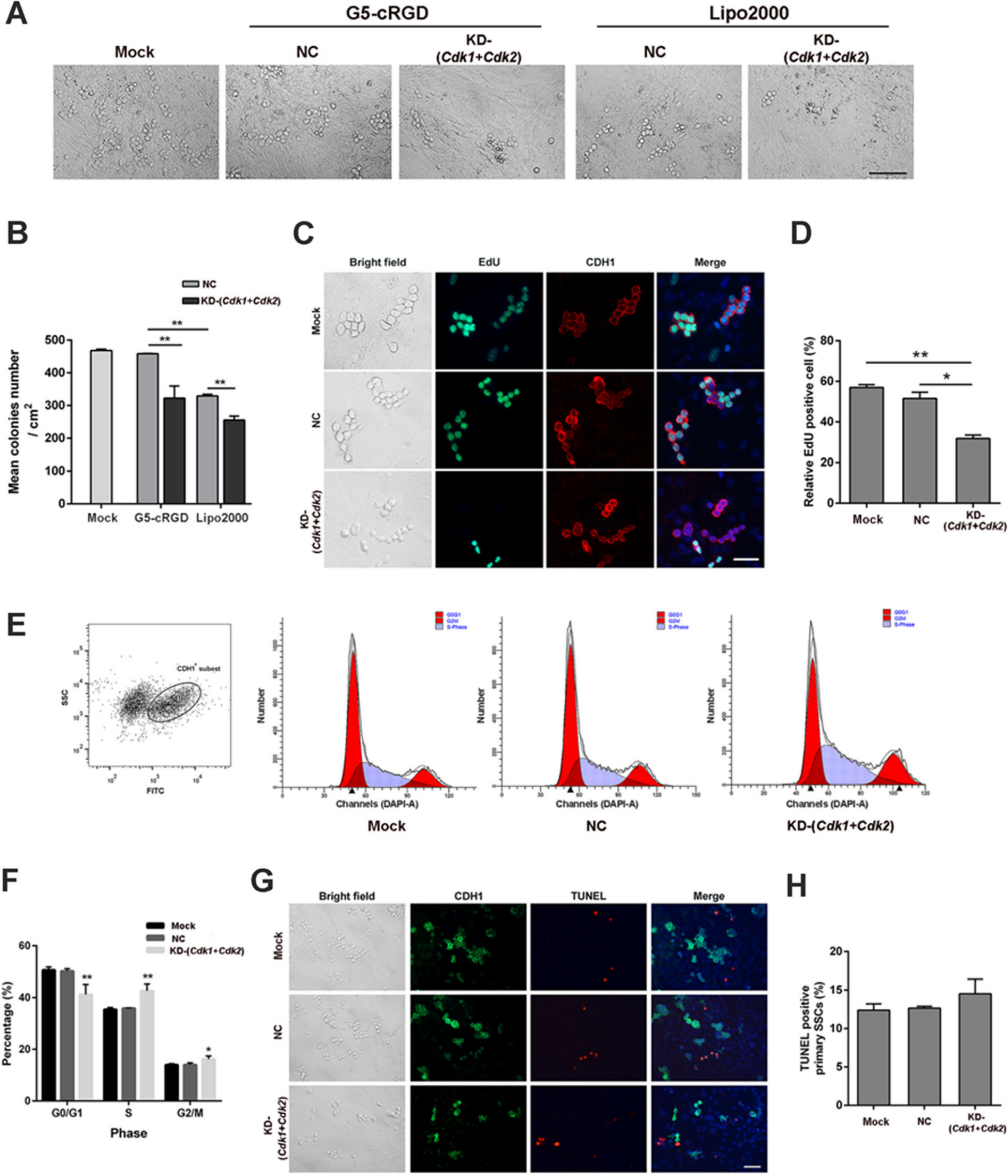
Functional siRNA delivery mediated by G5-cRGD leads to effective anti-proliferative effects in primary SSCs. **a** Appearance of cell colonies on day 5 after transfection with G5-cRGD-siRNA complexes. Lipo2000 was used as a control. Scale bars: 100 μm. **b** The mean number of colonies (a cluster consists of more than 5 cells) per square centimeter as shown in (**a**). **c** The EdU proliferation assay was performed 7 days after transfection with scrambled siRNA or *Cdk1*+*Cdk2* siRNA by G5-cRGD in primary SSCs. Red: CDH1 used to stain germ cell; green: EdU-positive cell; blue: Hoechst 33342 used to stain nuclei. Scale bars: 50 μm. **d** Ratios of EdU-positive germ cells to CDH1-positive cells. **e** Flow cytometry analysis of cell cycle of CDH1^+^ cells was performed 7 days after transfection with scrambled siRNA or *Cdk1*+*Cdk2* siRNA by G5-cRGD. **f** Quantitation of the cell cycle of the Mock, NC, or KD-(*Cdk1+Cdk2*) group cells as shown in (**e**). TUNEL assay was presented by (**g**) fluorescence microscope images and (**h**) TUNEL-positive cell counting (%). Red: TUNEL-positive cell; green: CDH1 was used to stain germ cell; DAPI used to stain nuclei. Scale bars: 50 μm. Statistical significance was assessed using one-way ANOVA followed by Dunnett’s test. Data are presented as mean ± standard deviation (SD, *n* = 3). *p < 0.05, **p < 0.01. Mock: untreated group; NC: scramble siRNA group; KD-(*Cdk1+Cdk2*): *Cdk1+Cdk2* siRNA group

Finally, knockdown of both *Cdk1* and *Cdk2* was examined in the primary SSCs. Compared with the NC group, delivery of siRNAs by G5-cRGD nanoparticles resulted in significant suppression of *Cdks* expression (Fig. 6d, e). We further quantified the SSC-derived colonies as an index for SSC proliferative activity. The average number of colonies in siRNA groups was significantly lower than those in NC groups when G5-cRGD or Lipo2000 was used as carriers (Fig. 7a). The significant reduction was observed in the G5-cRGD group (Fig. 7a, b). The Lipo2000-mediated NC group significantly reduced the number of colonies, while the G5-cRGD-mediated NC group was comparable to the Mock group, indicating G5-cRGD is less cytotoxic to SSCs. Subsequently, EdU assay showed that 31.84 ± 1.65% of the cells were positive for EdU staining in G5-cRGD group, while 57.10 ± 1.42% and 51.53 ± 3.12% EdU^+^ cells in the Mock and NC groups, respectively (Fig. 7c, d). Next, we investigated the cell cycle progression by DAPI/FACS. As shown in Fig. 7e, f, delivery of *Cdk1* and *Cdk2* siRNAs with G5-cRGD led to a significant increase in percentages of S and G2/M cell phases compared with the Mock and NC groups. As cell cycle arrest may cause apoptosis, we further explored the effect of CDK1 and CDK2 knockdown on apoptosis by TUNEL. The results demonstrated that 7 days after transfection, the TUNEL-positive cell rates in Mock, NC, and KD-(*Cdk1+Cdk2*) groups were 12.38 ± 0.83%, 12.66 ± 0.23%, 14.53 ± 1.89%, respectively (Fig. 7g, h). Compared with the Mock and NC groups, there was no significant difference in the KD-(*Cdk1+Cdk2*) group on apoptosis. In addition, no visible change in cell morphology was observed in cells transfected with scrambled siRNA/G5-cRGD, excluding the possibility of toxic effects of G5-cRGD. Therefore, G5-cRGD exhibits low cytotoxicity and high efficiency for functional delivery of siRNAs in the primary SSCs.

## Discussion

PAMAM dendrimers are the extensively studied non-viral vectors for siRNA delivery [13, 31]. SSCs are refractory to transfection, prompting us to probe whether PAMAM dendrimers can be used to efficiently deliver siRNAs to SSCs. Here, we demonstrated that G5-cRGD improved transfection efficiency and gene silencing in male germline stem cells.

Previous studies showed that the delivery efficiency of nanocomplexes was determined by numerous factors including generation, gene binding capacity, size, and toxicity [32]. It has been reported that lower generation dendrimers (G < 4) have an open structure, but dendrimers (G ≥ 4) possess a densely packed surface in higher generation [33]. Higher generation dendrimers are associated with more toxicity. The fifth generation of PAMAM dendrimers possesses lower toxicity and higher delivery capacity [34]. Their particle size is an important factor for cellular entry. A previous study reported that the optimal particle size for gene delivery via non-targeting cationic vector-DNA complexes is 70–90 nm [35]. In the present study, the mean diameter of dendriplexes was 70 nm. Meanwhile, cellular toxicity is another challenge for application in biological system. The cationic surface of dendrimer is inevitably adverse to the stability of cell membranes that are negatively charged [36]. Fortunately, several studies have revealed that the overall toxicity can be mitigated by surface modification with agents such as cRGD, PEG, and cyclodextrins [17, 37, 38]. Therefore, we modified G5 surfaces with cRGD and synthesized G5-cRGD nanocarrier. We found that this modification led to a significant reduction in cytotoxicity. One possible explanation is that cRGD could partially reduce the terminal cationic density. Taken together, these observations suggest that G5-cRGD may be a more efficient delivery system.

Previous studies reported that the efficiency of gene silencing was siRNA-concentration-dependent, and reached a plateau at more than 100 nM of siRNA [39]. In 2018, Ding et.al reported that gene silencing dramatically increased when concentration of the loaded siRNAs increased from 0 to 100 nM [40]. Several studies have revealed that 100 nM siRNA possessed high silencing efficiency [41, 42]. Therefore, we have chosen 100 nM siRNA in this work for transfection.

SSCs are a cornerstone of sperm production. However, studies and potential applications of SSCs have been greatly hampered by their low transfection efficiency. In this study, G5 modification with cRGD not only reduced the cytotoxicity but also improved transfection efficiency in primary SSCs, raising the possibility of a receptor-mediated endocytosis manner by cRGD-integrin recognition. Integrins are able to modulate interactions between cells and the extracellular matrix by specific recognition of Arg-Gly-Asp (RGD) peptide sequence [43]. As integrins are highly expressed in SSCs [44], it makes sense that cRGD modification would improve transfection efficiency. Moreover, the cRGD-dendriplexes internalization in an SSC line can occur at 37 °C, and to a lesser extent at 4 °C or NaN_3_, suggesting that cRGD-dendriplexes internalization is chiefly energy-dependent, but a passive translocation mechanism is not completely excluded.

Indeed, high transfection efficiency does not ensure powerful gene silencing. When nano-siRNA complexes cross the cell membrane, they are mainly enwrapped in acidic vehicles such as endosomes and lysosomes. To elicit RNA interference (RNAi), siRNAs need to be released from endosomes into cytoplasm. The inside tertiary amines confer PAMAM dendrimers efficiently, facilitating the intracellular release of siRNA by the “proton sponge” effect [45]. In this study, G5-cRGD displayed higher escape ability than the original G5, indicating that cRGD-dendriplexes were more effective for siRNAs to escape from endosomes. These results suggest that G5-cRGD may promote the endosomal escape of cRGD-dendriplexes and enhance silencing efficiency. To evaluate the delivery efficiency for gene silencing, we further co-delivered siRNAs against *Cdk1* and *Cdk2* that are pivotal cell cycle-regulatory molecules [46]. G5-cRGD significantly improved efficacy of gene silencing compared with the original G5. Here we demonstrated that G5-cRGD possessed high transfection efficiency, low cytotoxicity, and excellent endosomal escape ability, superior to a commercial transfection agent Lipofectamine 2000. Importantly, G5-cRGD-mediated siRNAs delivery led to downregulation of expression of *Cdk1* and *Cdk2*, and thus a decrease in the number of proliferating cells and SSC colonies. These results are in line with previous report in tumor cells [47]. Therefore, these observations demonstrate that G5-cRGD is a promising siRNA delivery vehicle in SSCs.

Moreover, this G5-cRGD delivery system is modular and can be modified to test other genes of interest. As more promising targets for SSCs emerge, this delivery platform is poised to provide an efficient method to rapidly validate new candidate genes in primary culture SSCs. Finally, the G5-cRGD nanoparticles are also a promising delivery for other stem cells and cancer cells in which integrins are highly expressed [48].

In spite of the development, we still need to investigate the clinical application in future studies. Indeed, one needs to realize that G5-cRGD nanoparticles are prone to degrade or interact with a variety of biological proteins in the extracellular environment. Another issue is that G5-cRGD delivery system could only load small RNAs or DNA fragments. It is not likely to load large fragment plasmids, such as CRISPR-Cas9 plasmids, and thus, we need to improve its loading efficiency. In addition, currently, there are many causes of a male factor-induced subfertility, including genomic mutation. However, although it is not applicable in clinics at present, G5-cRGD delivery system exhibits potential for further clinical applications.

## Conclusion

In summary, cRGD-conjugated G5 offer the advantages of nanoscale size and low toxicity, thereby holding the prospect to serve as an efficient vehicle for siRNA delivery in refractory cells including primary SSCs. Moreover, G5-cRGD-mediated delivery is safe for clinics, averting the risk of integration into the genome. In this sense, this study paves the way for future development and application of SSC auto-transplantation with genetically modified cells to clinics, with the hope of curing male infertility caused by genetic disorders.

## Supplementary information

**Additional file 1: Figure S1.** Representative of the Fourier transform infrared spectroscope (FTIR) spectrum for G5-NH_2_ and G5-cRGD. **Figure S2.** Characterization of the SSC line C18-4 cells. **Figure S3.** Fluorescent microscope images of an SSC line from the cellular uptake pathway experiments. **Figure S4.** Endosomal escape observed by CLSM. **Figure S5.** Characterization of primary Sertoli cells.

## Data Availability

All data generated or analyzed during this study are included in this published article and its additional information files.

## Abbreviations

SSCs: Spermatogonial stem cells
PAMAM: Poly(amidoamine)
G5: The fifth generation of PAMAM dendrimers
cRGD: Cyclic arginine-glycine-aspartic acid
G5-cRGD: PAMAM-cRGD dendrimers
FTIR: Fourier transform infrared spectroscope
TEM: Transmission electron microscope
CCK-8: Cell Counting Kit-8
siRNAs: Short interfering RNAs
Lipo2000: Lipofectamine® 2000
Oct4: Octamer-binding transcription factor 4
FACS: Fluorescent Activated Cell Sorting
KSR: KnockOut Serum Replacement
MEM: Minimal essential medium
GDNF: Glial cell line-derived neurotrophic factor
ICR: Institute of Cancer Research
EDC: 1-Ethyl-3-(3-dimethylaminopropyl) carbodiimide hydrochloride
NHS: *N*-Hydroxysuccinimide
Cdk1: Cyclin-dependent kinase 1
Cdk2: Cyclin-dependent kinase 2
NC: Scrambled siRNA
PBS: Phosphate buffer saline
MwCO: Molecular weight cut off
KBr: Potassium bromide
EDTA: Ethylenediaminetetraacetic acid
DMEM: Dulbecco’s modified Eagle medium
FBS: Fetal bovine serum
bFGF: Basic fibroblast growth factor
N/P ratio: The ratio of nitrogen atoms in the dendrimer to phosphorous atoms in the siRNA
Dendriplexes: G5-siRNA complexes
cRGD-dendriplexes: G5-cRGD-siRNA complexes
UV: Ultraviolet
NP: Nanoparticle
FAM: 6-Carboxy-fluorescein
MFI: Mean fluorescence intensity
GFP: Green fluorescent protein
NaN_3_: Sodium azide
CLSM: Confocal laser scanning microscope
EdU: 5-Ethynyl-2′-deoxyuridine
TUNEL: TdT-mediated dUTP Nick-End Labeling
DAPI: 4′,6-Diamidino-2-phenylindole
PFA: Paraformaldehyde
THY1: Thymocyte antigen 1
CDH1: E-cadherin
Lin28: Lin-28 homolog A
PCNA: Proliferating cell nuclear antigen
PLZF: Promyelocytic leukemia zinc-finger
PEG: Polyethylene glycol
