# Supplementary material for: PAMAM-cRGD mediating efficient siRNA delivery to spermatogonial stem cells
Source: Stem Cell Res Ther. 2019 Dec 18;10:399. doi: 10.1186/s13287-019-1506-4 (PMC6921429; doi:10.1186/s13287-019-1506-4)
Supplement: Supplementary file 1 — Additional file 1: Figure S1. Representative of the Fourier transform infrared spectroscope (FTIR) spectrum for G5-NH2 and G5-cRGD. Figure S2. Characterization of the SSC line C18-4 cells. Figure S3. Fluorescent microscope images of an SSC line from the cellular uptake pathway experiments. Figure S4. Endosomal escape observed by CLSM. Figure S5. Characterization of primary Sertoli cells. [file 13287_2019_1506_MOESM1_ESM.docx]

Supporting Information

**PAMAM-cRGD mediating efficient siRNA delivery to spermatogonial stem cells**

Tianjiao Li ^1, 2, 3, #^, Qiwen Chen ^4, #^, Yi Zheng ^1, 2, 3^, Pengfei Zhang ^1, 2, 3^, Xiaoxu Chen ^1, 2, 3^, Junna Lu ^4^, Yinghua Lv ^1, 4^, Shiguo Sun^4, *^, Wenxian Zeng ^1, 2, 3, *^

1 College of Animal Science and Technology, Northwest A&F University, Yangling, Shaanxi, 712100, China

2 Key Laboratory of Animal Biotechnology, Ministry of Agriculture, Northwest A&F University, Yangling, Shaanxi, 712100, China

3 Key Laboratory of Animal Genetics, Breeding and Reproduction of Shaanxi Province, Northwest A&F University, Yangling, Shaanxi, 712100, China

4 Shaanxi Key Laboratory of Natural Products & Chemical Biology, College of Chemistry & Pharmacy, Northwest A&F University, Yangling, Shaanxi, 712100, China.

# These authors contributed equally to this work as co-first authors.

* Corresponding authors: Wenxian Zeng ([zengwenxian2015@126.com](mailto:zengwenxian2015@126.com)), Shiguo Sun (sunsg@nwsuaf.edu.cn)


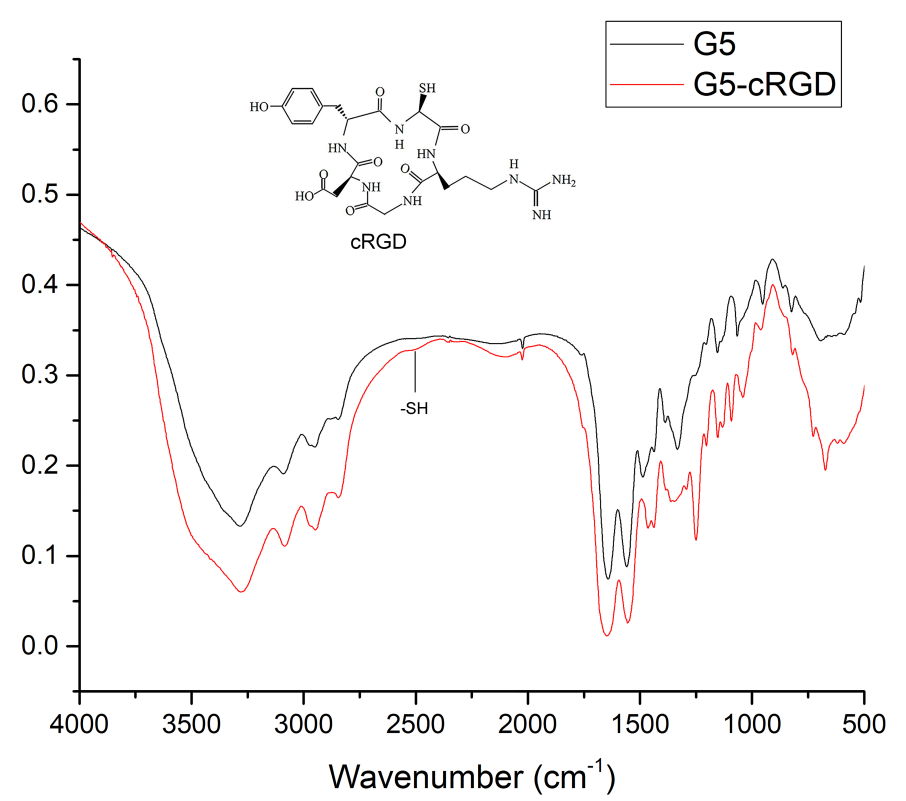


**Figure S1**

Representative of the Fourier transform infrared spectroscope (FTIR) spectrum for G5-NH_2_ and G5-cRGD.


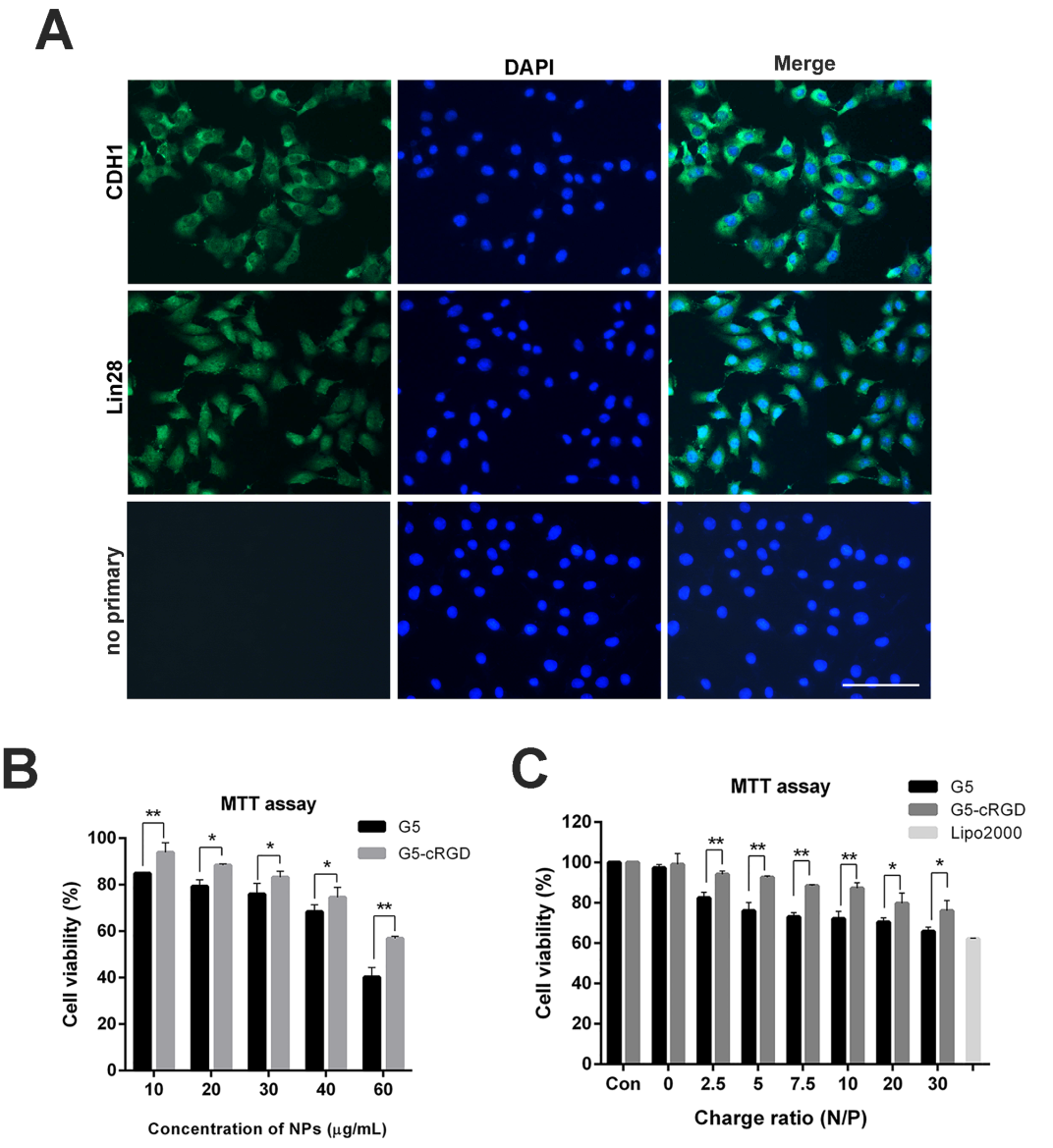


**Figure S2**

Characterization of the SSC line C18-4 cells. (A) Immunocytochemical staining showing the expression of CDH1 (green fluorescence) and Lin28 (green fluorescence) in the C18-4 cells. Negative (no primary) control: omission of primary antibody. The nuclei (blue) were stained with DAPI. The scale bar is 100 µm. (B) The viability of SSCs incubated with G5 and G5-cRGD nanoparticles at various concentrations by MTT assay. (C) The viability of SSCs transfected with G5-siRNA and G5-cRGD-siRNA complexes at different N/P ratios for 24 h, evaluated by MTT assay. The final siRNA concentration of each sample was 100 nM. Lipo2000 was used as a positive control. Statistical significance was determined by applying the Student’s *t* test. Data are presented as mean ± standard deviation (SD, n = 3). **p* < 0.05, ***p* < 0.01.


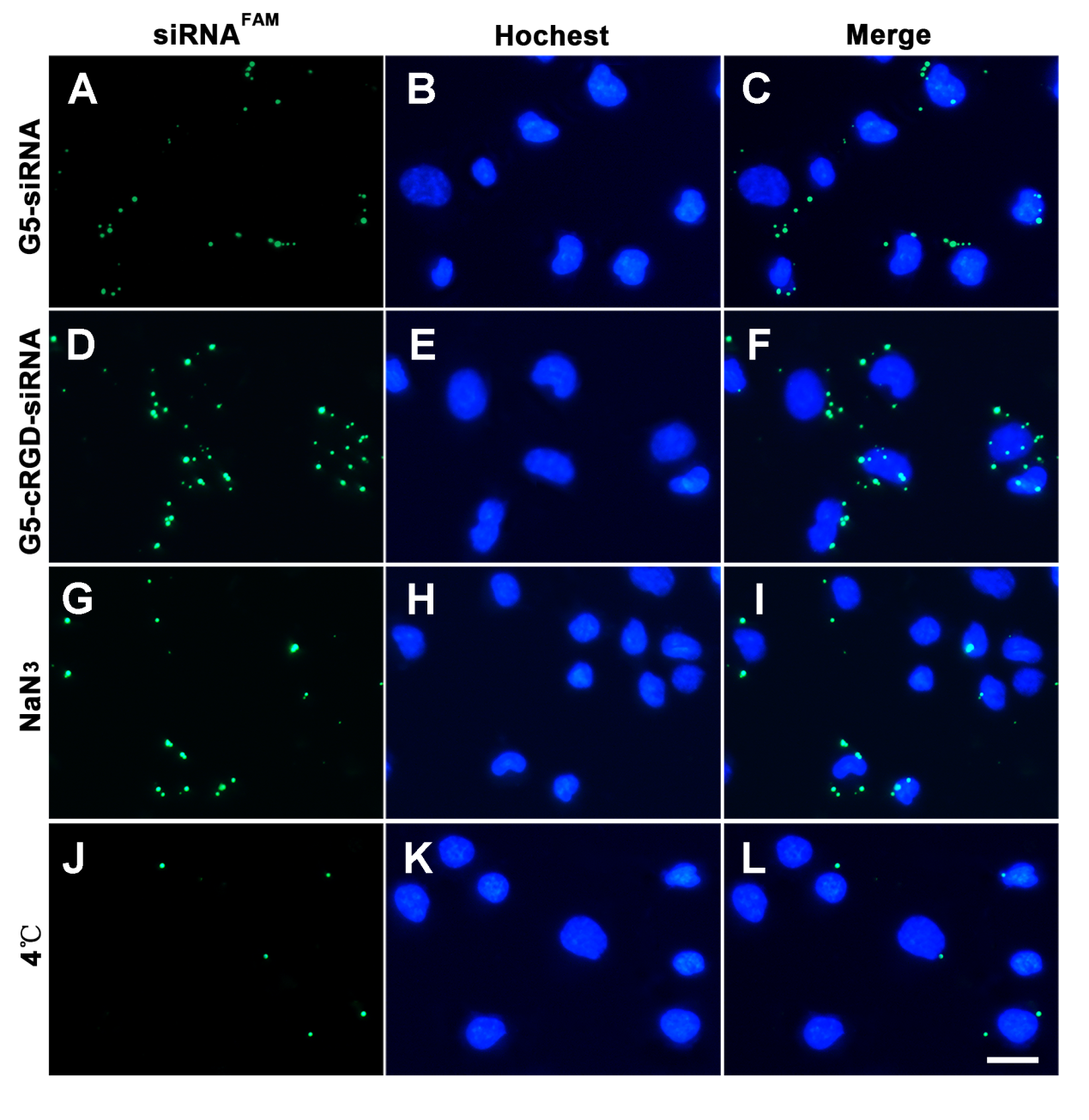


**Figure S3**

Fluorescent microscope images of an SSC line from the cellular uptake pathway experiments. Scale bars: 20 µm.


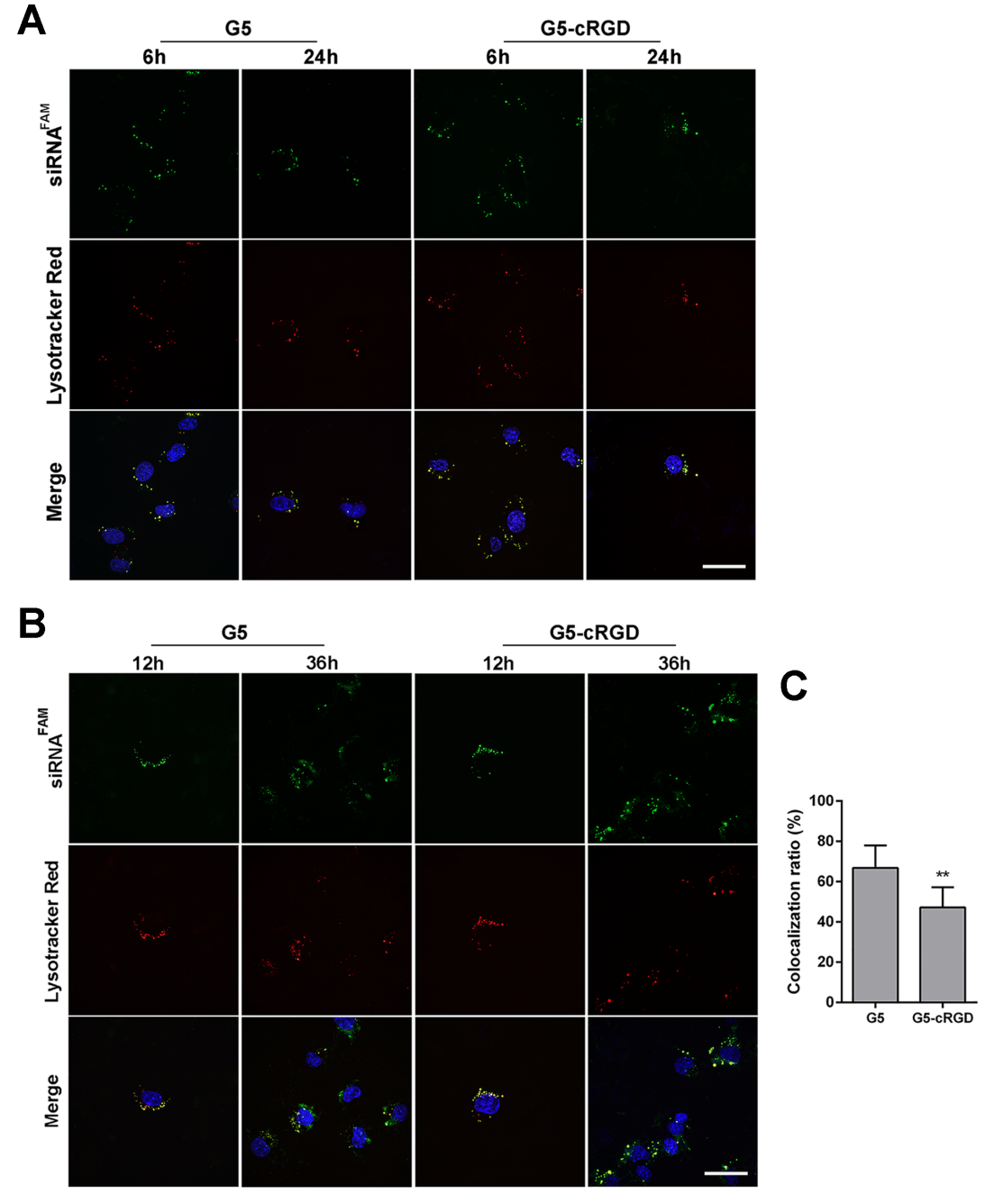


**Figure S4**

Endosomal escape observed by CLSM. (A) Confocal images were taken after C18-4 cells were transfected with G5-cRGD-siRNA or G5-siRNA complexes at 6 or 24 h. (B) Confocal images were taken after C18-4 cells were transfected with G5-cRGD-siRNA or G5-siRNA complexes at 12 or 36 h. The nuclei (blue) were stained with Hoechst 33342. The green signal comes from the FAM-labeled siRNA, while the late endosome and lysosome are stained with LysoTracker Red (red fluorescence). Yellow emission indicates co-localization. The scale bar is 20 µm. (C) Co-localization ratio of FAM-labeled siRNA with LysoTracker is calculated by counting puncta at 36 h (number of counted cells: 100). Statistical significance was determined by applying the Student’s *t* test. Data are presented as mean ± standard deviation (SD). **p* < 0.05, ***p* < 0.01.


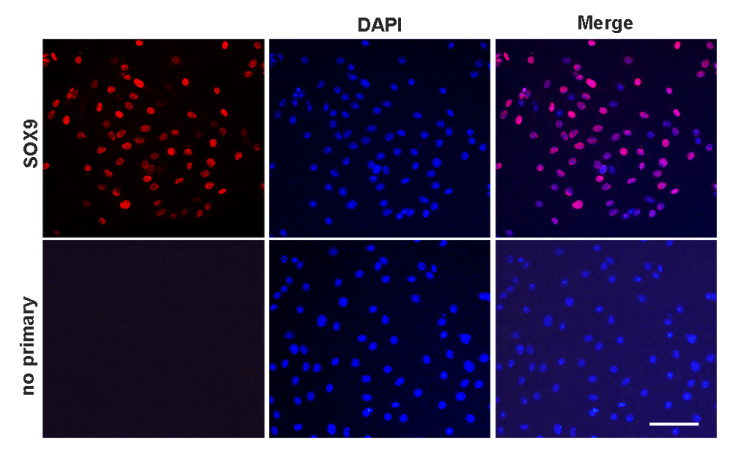


**Figure S5**

Characterization of primary Sertoli cells. Immunocytochemical staining showing the expression of SOX9 (red fluorescence) in primary Sertoli cells. Negative (no primary) control: omission of primary antibody. The nuclei (blue) were stained with DAPI. The scale bar is 100 µm.
